# Vitamin D_3_ Supplementation Reduces Subsequent Brain Injury and Inflammation Associated with Ischemic Stroke

**DOI:** 10.1007/s12017-018-8484-z

**Published:** 2018-02-23

**Authors:** Megan A. Evans, Hyun Ah Kim, Yeong Hann Ling, Sandy Uong, Antony Vinh, T. Michael De Silva, Thiruma V. Arumugam, Andrew N. Clarkson, Graeme R. Zosky, Grant R. Drummond, Brad R. S. Broughton, Christopher G. Sobey

**Affiliations:** 1 Vascular Biology Immunopharmacology Group, Department of Physiology, Anatomy and Microbiology, La Trobe University, Bundoora, VIC 3083 Australia; 2 Cardiovascular Disease Program, Department of Pharmacology, Biomedicine Discovery Institute, Monash University, Clayton, VIC 3800 Australia; 3 Department of Physiology, Yong Loo Lin School of Medicine, National University of Singapore, Singapore, 119228 Singapore; 4 School of Pharmacy, Sungkyunkwan University, Suwon, South Korea; 5 Department of Anatomy, Brain Health Research Centre and Brain Research New Zealand, University of Otago, Dunedin, 9054 New Zealand; 6 School of Life Sciences, La Trobe University, Bundoora, VIC 3083 Australia; 7 School of Medicine, Faculty of Health Science, University of Tasmania, Hobart, TAS 7005 Australia

**Keywords:** Vitamin D, Inflammation, Stroke, Middle cerebral artery occlusion, Mouse

## Abstract

Acute inflammation can exacerbate brain injury after ischemic stroke. Beyond its well-characterized role in calcium metabolism, it is becoming increasingly appreciated that the active form of vitamin D, 1,25-dihydroxyvitamin D_3_ (1,25-VitD_3_), has potent immunomodulatory properties. Here, we aimed to determine whether 1,25-VitD_3_ supplementation could reduce subsequent brain injury and associated inflammation after ischemic stroke. Male C57Bl6 mice were randomly assigned to be administered either 1,25-VitD_3_ (100 ng/kg/day) or vehicle i.p. for 5 day prior to stroke. Stroke was induced via middle cerebral artery occlusion for 1 h followed by 23 h reperfusion. At 24 h post-stroke, we assessed infarct volume, functional deficit, expression of inflammatory mediators and numbers of infiltrating immune cells. Supplementation with 1,25-VitD_3_ reduced infarct volume by 50% compared to vehicle. Expression of pro-inflammatory mediators IL-6, IL-1β, IL-23a, TGF-β and NADPH oxidase-2 was reduced in brains of mice that received 1,25-VitD_3_ versus vehicle. Brain expression of the T regulatory cell marker, Foxp3, was higher in mice supplemented with 1,25-VitD_3_ versus vehicle, while expression of the transcription factor, ROR-γ, was decreased, suggestive of a reduced Th17/γδ T cell response. Immunohistochemistry indicated that similar numbers of neutrophils and T cells were present in the ischemic hemispheres of 1,25-VitD_3_- and vehicle-supplemented mice. At this early time point, there were also no differences in the impairment of motor function. These data indicate that prior administration of exogenous vitamin D, even to vitamin D-replete mice, can attenuate infarct development and exert acute anti-inflammatory actions in the ischemic and reperfused brain.

**Electronic supplementary material:**

The online version of this article (10.1007/s12017-018-8484-z) contains supplementary material, which is available to authorized users.

## Introduction

Stroke is the world’s second leading cause of death, contributing to 6.7 million deaths annually (Mozaffarian et al. 2016). It is also the most frequent cause of permanent disability in adults, with half of all survivors discharged into care (Mozaffarian et al. 2016). Currently, there is only one approved pharmacological agent available to treat stroke, recombinant tissue plasminogen activator (rtPA), which must be administered within a 4.5-h window of stroke onset and only after a CT scan has diagnosed a thrombotic cause (Del Zoppo et al. 2009). Due to these strict limitations, < 10% of stroke patients are eligible to receive rtPA (Reeves et al. 2005; Kleindorfer et al. 2008). Consequently, there is a desperate need to identify modifiable mechanisms capable of limiting the impact of acute stroke.

Secondary brain injury following stroke is driven by local inflammation, production of reactive oxygen species and the infiltration of circulating immune cells (Anrather and Iadecola 2016). Thus, targeting these inflammatory processes has been of intense interest to stroke researchers. However, one immunomodulatory molecule that has received very little attention as a potential stroke therapy is vitamin D, a fat-soluble vitamin that functions as a steroid hormone. Vitamin D is synthesized predominantly from 7-dehydrocholesterol in response to skin exposure to ultraviolet light, but can also be obtained through dietary supplementation (Holick 2007). To become biologically active, vitamin D must first be converted to 1,25-dihydroxyvitamin D_3_ (1,25-VitD_3_) via two hydroxylation steps. This occurs firstly in the liver by 25-hydroxylase and then typically in the kidney by 1-α-hydroxylase (CYP27B) (Holick 2007). The latter hydroxylation step can also occur in macrophages, T cells and neurons, which also express 1-α-hydroxylase (Lugg et al. 2015). Once in this active form, vitamin D can engage with the vitamin D receptor (VDR) which is located on a number of cell types including leukocytes, endothelial cells, astrocytes and neurons (Provvedini et al. 1983; Merke et al. 1989; Langub et al. 2001; Lee et al. 2008). Vitamin D is best characterized to promote calcium absorption from the small intestine, but recent findings indicate that it may also control expression of a large number of genes, particularly those involved in inflammatory processes (Lugg et al. 2015).

1,25-VitD_3_ exerts such immunomodulatory actions through a variety of cellular and molecular mechanisms. Firstly, 1,25-VitD_3_ can prevent the development of pathogenic T helper (Th) 1, Th17 and γδ T cells, and can promote the formation of anti-inflammatory Th2 and T regulatory cells (Zeitelhofer et al. 2017; Chang et al. 2010a; Gregori et al. 2002; Joshi et al. 2011; Nashold et al. 2013; Sloka et al. 2011; Cantorna et al. 2004; Hart et al. 2011; Chen et al. 2005). Studies have also shown that 1,25-VitD_3_ promotes the generation of tolerogenic dendritic cells (Takeda et al. 2010; Gorman et al. 2010) and can prevent the release of pro-inflammatory cytokines from monocytes and microglia (Korf et al. 2012; Zhang et al. 2012; Boontanrart et al. 2016; Verma and Kim 2016). Further, 1,25-VitD_3_ may inhibit the production of reactive oxygen species by decreasing expression of NADPH oxidase (NOX) enzymes (Dong et al. 2012) and enhancing expression of antioxidants such as superoxide dismutase and glutathione (Jain and Micinski 2013; Dong et al. 2012).

Observational studies have documented that patients with lower serum levels of vitamin D experience larger infarct volumes and worse functional outcomes following stroke (Tu et al. 2014; Wang et al. 2014; Turetsky et al. 2015; Daubail et al. 2013; Park et al. 2015), suggesting that vitamin D may play a protective role during cerebral ischemia. We recently reported that low baseline levels of vitamin D, resulting from a vitamin D-deficient diet, had no discernible impact on selected outcome measures within 24 h of large vessel occlusion stroke (Evans et al. 2017). Here, we have instead examined the effect of elevated baseline levels of vitamin D achieved by supraphysiological doses of vitamin D given to vitamin D-replete animals during the 5 days prior to stroke, in an analogous manner to high dose supplementation regimes in humans (Wong et al. 2014; Sotirchos et al. 2016). For this, we adopted a similar supplementation regime that was found to reduce vascular injury in mice following hindlimb ischemia (Wong et al. 2014). Indeed, we report that 1,25-VitD_3_ supplementation can reduce post-stroke brain injury, reduce expression of pro-inflammatory cytokines, modulate the phenotype of T cells and increase the number of M2-polarized (anti-inflammatory) macrophages/microglia in the brain.

## Materials and Methods

### Animals

A total of 92 male C57Bl6 mice (7–10 week old; 21–30 g) were used for this study. Mice were housed under a 12-h light/dark cycle and had free access to water and food pellets. Mice were excluded from the study if during the surgical procedure to induce middle cerebral artery occlusion: [1] > 0.2 ml of blood was lost (*n* = 1); [2] subarachnoid hemorrhage occurred (*n* = 2); [3] death occurred during ischemia (*n* = 2); [4] cerebral blood flow failed to reach ≥ 80% pre-ischemic levels upon reperfusion (*n* = 1) and [5] death occurred after reperfusion and prior to the designated time for euthanasia (*n* = 7). All animals were randomly assigned to groups, and the investigator performing the surgical procedure or data analysis was, wherever possible, blinded to the treatment group.

### Administration of 1-25,Dihydroxyvitamin D_3_

Vitamin D_3_ was administered as its active form, 1α, 25-dihydroxyvitamin D_3_ (1,25-VitD_3_; Sigma; 100 ng/kg/day) which was dissolved in a solvent mixture of sterile water, propylene glycol and ethanol in a 5:4:1 ratio. Animals were injected i.p. for 5 consecutive days prior to experimental stroke and again on the day of the procedure, as previously described (Wong et al. 2014).

### Middle Cerebral Artery Occlusion

Mice underwent either sham surgery or focal cerebral ischemia as previously described (Evans et al. 2017). Cerebral ischemia was produced in anesthetized mice (ketamine: 80 mg/kg plus xylazine: 10 mg/kg i.p.) by occlusion of the middle cerebral artery (MCA) using a 6.0 silicone-coated monofilament (Doccol Corporation). Rectal temperature was monitored and maintained at 37.0 ± 0.5 °C. MCA occlusion (MCAO) was sustained for 60 min, and the filament then retracted to allow reperfusion. Both successful occlusion (> 70% reduction in cerebral blood flow; CBF) and reperfusion (≥ 80% return of CBF to the pre-ischemic level) were confirmed by transcranial laser-Doppler flowmetry (PeriMed). Sham-operated mice were anesthetized and the right carotid bifurcation exposed, but no filament was inserted. Neck wounds were then closed with sutures and covered with Betadine^®^ (Sanofi) and spray dressing. Head wounds were closed with superglue, and mice were returned to their cages after regaining consciousness.

### Functional Assessment

Mice were assessed for functional deficits at approximately 30 min prior to euthanasia. This comprised a 6-point scoring system for neurological deficits: 0 = normal motor function, 1 = flexion of torso and contralateral forelimb when lifted by the tail, 2 = circling to the contralateral side when held by the tail on a flat surface but normal posture at rest, 3 = leaning to the contralateral side at rest, 4 = no spontaneous motor activity, 5 = death. A hanging grip test was performed as a measure of grasping ability and forelimb strength in which mice were suspended by their forelimbs on a wire between 2 posts 60 cm above a soft pillow for up to 60 s. The time until the animal fell was recorded (a score of 0 s was assigned to animals that fell immediately and a score of 60 s was assigned to animals that did not fall), and the average time of 3 trials with 5 min rests in between was calculated. Spontaneous locomotor activity was assessed using a parallel rod floor apparatus using ANY-maze software coupled to an automated video-tracking system as previously described (Lee et al. 2015).

### Assessment of Infarct Volume

Cerebral infarct volumes were determined as previously described (Evans et al. 2017). At 24 h post-stroke, mice were killed by inhalation of isoflurane followed by decapitation. Brains were immediately removed, snap-frozen in liquid nitrogen and stored at − 80 °C. Evenly spread (separated by ~ 420 μm) coronal sections (30 μm) spanning the infarct were cut, thaw-mounted onto poly-l-lysine coated glass slides and stained with 0.1% thionin (Sigma) to delineate the infarct area. Infarct volume was quantified using image analysis software (ImageJ, NIH), correcting for brain edema, according to the following formula: CIV = (LHA − (RHA − RIA)) × (thickness of section + distance between sections); where *CIV* is corrected infarct volume, *LHA* is left hemisphere area, *RHA* is right hemisphere area and *RIA* is right hemisphere infarct area. Edema-corrected infarct volumes of individual brain sections were then added, giving an approximation of the total infarct volume.

### Real-Time Polymerase Chain Reaction (rt-PCR)

At 24 h following stroke or sham surgery, mice were euthanized by isoflurane overdose and perfused with RNase-free phosphate-buffered saline (PBS). After removing the cerebellum and olfactory bulbs, the brain was separated into left and right hemispheres and snap-frozen in liquid nitrogen for RNA extraction. Spleens were also removed, cut in half and snap-frozen in liquid nitrogen. Tissues were stored at − 80 °C until required. Total RNA was extracted using Qiazol^®^ reagent (Qiagen) and the RNeasy Mini Kit with on-column DNase step (Qiagen) followed by cDNA conversion using the Quantitect Reverse Transcription kit (for Taqman^®^ gene expression assays; Qiagen). The cDNA was then used as a template in real-time PCR to measure mRNA expression of *Vdr*, *Cyp27b*, *Cyp24a*, *Cxcl12*, *Tbx21*, *Stat4*, *Rorc*, *Gata3*, *Stat6*, *Foxp3*, *Tnfα*, *Il1β*, *Il6*, *Il21*, *Il23a*, *Tgfβ1*, *Ccl2*, *Ccl5*, *Gp91phox*, *Mrc1*, *Il10* and *Icam1*. *Gapdh* and *β*-*actin* were assessed as housekeeping genes for brain and spleen tissue, respectively. Assays were performed according to the manufacturer’s instructions using the Bio-Rad CFX96TM real-time PCR machine (Bio-Rad). Data were normalized to the housekeeping gene and calculated as change in fold expression relative to sham using the formula: fold-change = 2^−ΔΔCt^.

### Immunofluorescence

Six serial coronal sections (10 μm thick) per animal were collected at six regions: bregma + 0.06, − 0.78, − 1.2, − 1.62, − 2.04, − 2.46 mm. Frozen brain sections (10 μm) were fixed in 4% paraformaldehyde for 15 min and washed in 0.01 M PBS (3 × 10 min). Sections were then blocked with 10% goat serum (Sigma) for 60 min to block non-specific binding of the secondary antibody. Sections were then incubated overnight at 4 °C with either rabbit anti-CD3 (1:200; Abcam) or rabbit anti-CD206 (1:500; Abcam). On the following day, they were washed (PBS; 3 × 10 min) and incubated for a maximum of 2 h with either goat anti-rabbit Alexa Fluor 594 (1:500; Thermofisher Scientific) or goat anti-rabbit Alexa Fluor 488 (1:500; Thermofisher Scientific). Finally, sections were again washed and then mounted with Vectashield medium containing 4,6-diamidino-2-phenylindole (DAPI) (Vector Laboratories), and a coverslip was applied. All tissue-mounted slides were viewed, analyzed and photographed with an Olympus fluorescence microscope. Numbers of immunoreactive cells were counted manually per whole ischemic hemisphere and then averaged across the six regions, as indicated above.

### 3,3′-Diaminobenzidine (DAB) Immunohistochemistry

Frozen brain sections (at the regions indicated for immunofluorescence) were fixed in 4% paraformaldehyde for 15 min, washed in PBS and then incubated in peroxidase blocking solution (Dako) for 10 min to block endogenous peroxidases followed by 10% goat serum for 60 min. They were then incubated overnight at room temperature in rabbit anti-myeloperoxidase (1:100; Abcam). The following day, sections were washed and incubated for 2 h in anti-rabbit IgG horse-radish peroxidase conjugate (1:200; Dako), washed again, and DAB (Dako) was then applied for 5–10 min. Sections were then rinsed in dH_2_O, dehydrated in increasing concentrations of ethanol (70 and 100% vol/vol), cleared in xylene and mounted in DPX. Tissue-mounted slides were viewed, analyzed and photographed using an Olympus light microscope. Numbers of immunoreactive cells were counted manually per whole ischemic hemisphere and then averaged across the six regions, as indicated above.

### Statistical Analysis

Data are presented as mean ± standard error of the mean (SEM), with the exception of neurological deficit scores, which are presented as median. Statistical analyses were performed using GraphPad Prism version 6.0 (GraphPad Software Inc. San Diego, CA, USA). Between-group comparisons were compared using one-way ANOVA, or Student’s unpaired *t* test, as appropriate. If differences were detected by ANOVA, individual groups were compared with Tukey’s multiple comparisons test, where indicated. Neurological deficit scores were compared using a Kruskal–Wallis test followed by Dunn’s multiple comparisons test. If there were two independent variables, data were compared using a two-way ANOVA. Statistical significance was accepted if *P* < 0.05.

## Results

### Effect of Cerebral Ischemia on Vitamin D-Associated Genes in Brain and Spleen

**Fig. 1:**
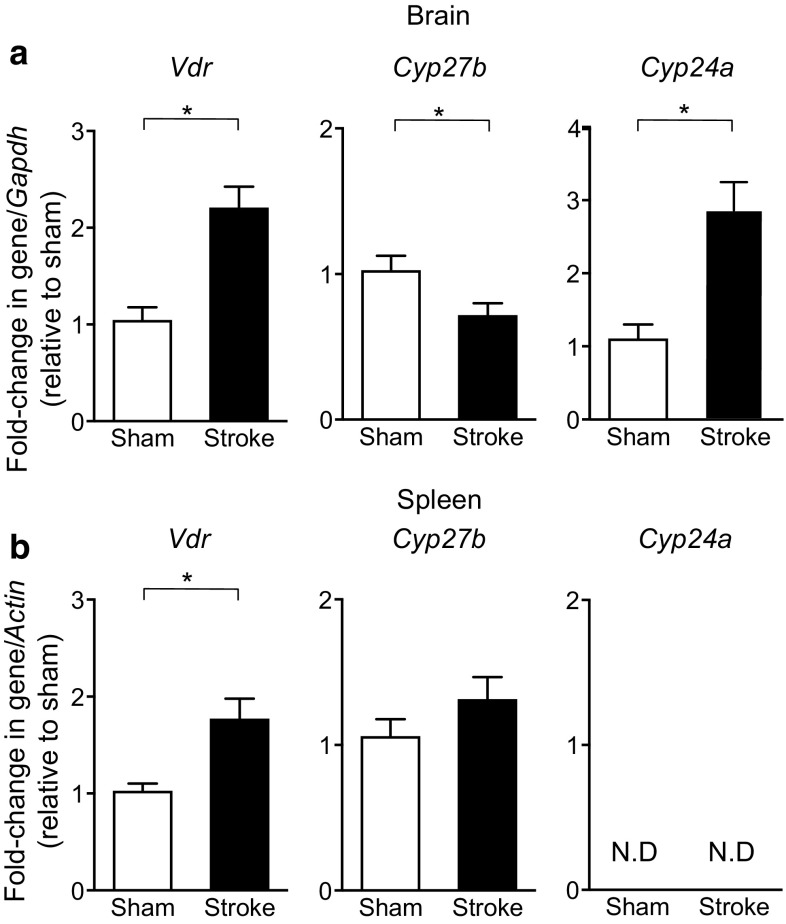
Post-stroke expression of vitamin D-associated genes. mRNA expression of the vitamin D receptor (*Vdr*), 1-α-hydroxylase (*Cyp27b*) and 24-hydroxylase (*Cyp24a*) was examined in **a** the brain and **b** the spleen at 24 h after either stroke or sham surgery. Sham: *n* = 6–9 per group and stroke: *n* = 8–12 per group. *N.D.* denotes not detected. *P < 0.05, Student’s unpaired *t* test. Data are presented as mean ± SEM

The effect of stroke was first assessed on the expression of VDR and metabolizing enzymes in the brain and spleen at 24 h in otherwise untreated animals. Stroke increased expression of the VDR by ~twofold in both organs (Fig. 1a, b). The vitamin D-activating enzyme*, Cyp27b*, was reduced by ~ 30% in the brain, but was unchanged in the spleen following stroke (Fig. 1a, b). Expression of the inactivating enzyme, *Cyp24a*, was increased by ~ 2.5 fold in the brain, but was undetectable in spleen (Fig. 1a, b).

### Effects of Vitamin D_3_ Supplementation on Infarct Volume and Functional Deficits Following Stroke

**Fig. 2:**
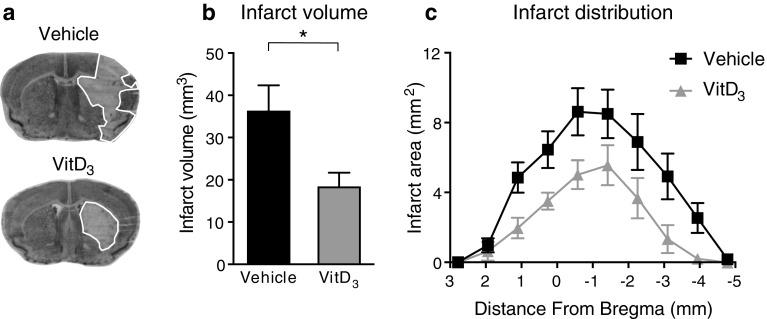
1,25-Dihydroxyvitamin D_3_ (VitD_3_) treatment reduces infarct development. **a** Representative coronal brain sections from vehicle- and VitD_3_-treated animals (infarct area is outline in white). **b** Infarct volume and **c** distribution of infarct area from vehicle- and VitD_3_-treated animals. Vehicle: *n* = 14 per group and VitD_3_: *n* = 12 per group. *P < 0.05, Student’s unpaired *t* test, where appropriate. Data are presented as mean ± SEM

**Fig. 3:**
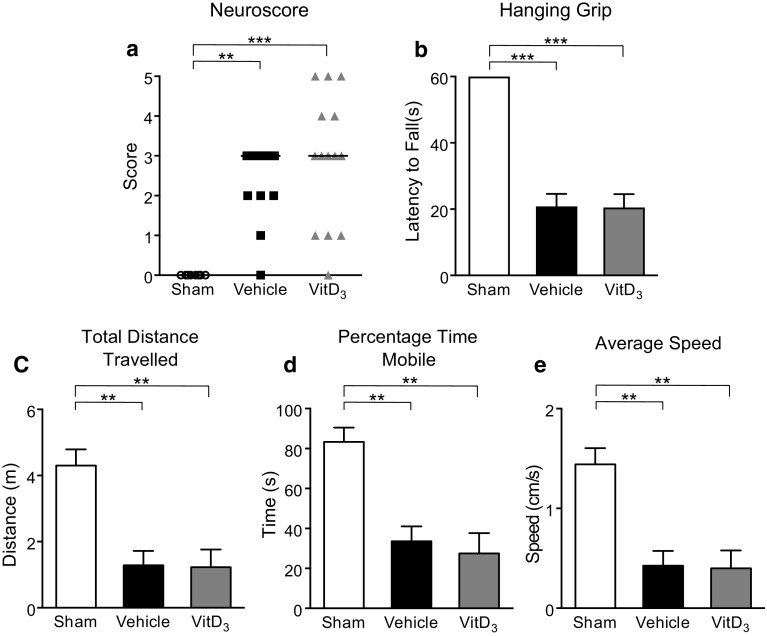
1,25-Dihydroxyvitamin D_3_ (VitD_3_) treatment does not influence functional outcome at 24 h following stroke. **a** Neurological deficit scores and **b** latency to fall on hanging grip test of sham, vehicle- and VitD_3_-treated animals. Parallel rod floor test examining **c** total distance travelled, **d** percentage time mobile and **e** average speed of sham, vehicle- and VitD_3_-treated animals. Sham: *n* = 6–8 per group, vehicle: *n* = 14 per group and VitD_3_: *n* = 11–14 per group. **P < 0.01, ***P < 0.001, Kruskal–Wallis test followed by Dunn’s multiple comparisons test (**a**) or one-way ANOVA followed by Tukey’s multiple comparison test (b–e). Data are presented as mean ± SEM, with the exception of neurological deficit scores which are presented as median

To determine the effect of elevated baseline vitamin D prior to stroke on the extent of subsequent infarct development, mice were treated with 1,25-VitD_3_ (100 ng/kg/day) for 5 days and then subjected to focal cerebral ischemia. At 24 h post-stroke, we found that mice which received 1,25-VitD_3_ supplementation had ~ 50% smaller infarct volume than those which received vehicle (Fig. 2a, b). This finding was not associated with any differences in the level of cerebral blood flow during, or immediately after, cerebral ischemia (Fig. S1). Examining the distribution of the infarcts, 1,25-VitD_3_-supplemented animals tended to have a reduced infarct area in most coronal sections (Fig. 2c). However, mice in both groups displayed similar functional deficits at this early time point (Fig. 3a–e).

### Effect of Vitamin D_3_ Supplementation on T Cell Phenotype in the Brain and Spleen After Stroke

**Fig. 4:**
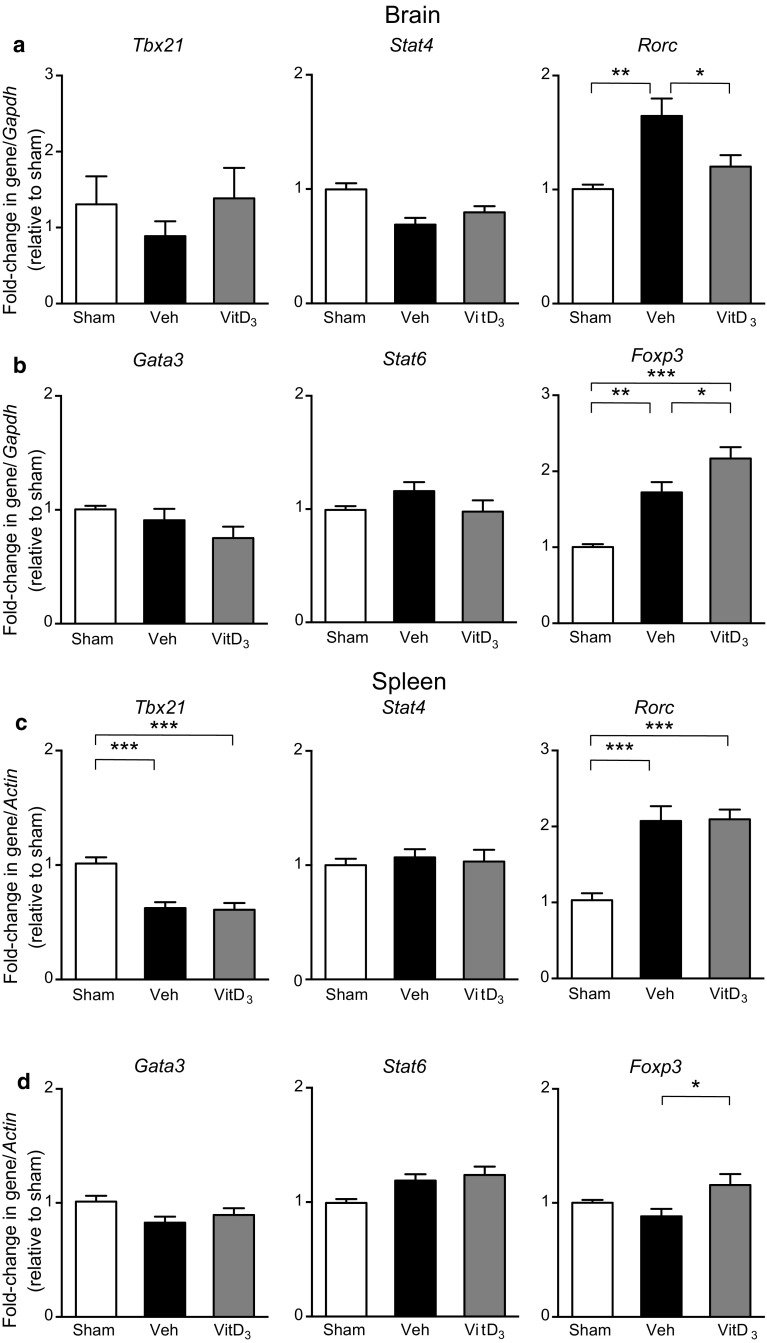
1,25-Dihydroxyvitamin D_3_ (VitD_3_) supplementation alters mRNA expression of T cell transcription factors in the brain and spleen following stroke. **a** Expression of Th1 transcription factors, *Tbx21* and *Stat4*, and Th17 transcription factor, *Rorc*, within the brain of sham, vehicle- (Veh) and VitD_3_-treated mice at 24 h post-surgery. **b** Expression of Th2 transcription factors, *Gata3* and *Stat6,* and T regulatory cell transcription factor, *Foxp3*, within the brain of sham, vehicle- and VitD_3_-treated mice at 24 h post-surgery. **c** Expression of *Tbx21*, *Stat4*, and *Rorc* within the spleen of sham, vehicle- and VitD_3_-treated mice at 24 h post-surgery. **d** Expression of *Gata3*, *Stat6* and *Foxp3* within the spleen of sham, vehicle- and VitD_3_-treated mice 24 h post-surgery. Sham: *n* = 6–9 per group, vehicle: *n* = 11–13 per group, VitD_3_: *n* = 10–12 per group. *P < 0.05, **P < 0.01, ***P < 0.001, one-way ANOVA followed by Tukey’s multiple comparisons test. Data are presented as mean ± SEM

Previous reports suggest that Th1 and γδ T cells exacerbate brain injury following stroke while Th2 and T regulatory cells play a protective role by dampening excessive inflammation (Gu et al. 2012; Gelderblom et al. 2012; Benakis et al. 2016; Liesz et al. 2009). It has been shown that vitamin D can modulate the immune response to injury by polarizing T cells toward an anti-inflammatory phenotype (Hart et al. 2011). We found no effect of stroke or 1,25-VitD_3_ on mRNA expression of Th1 transcription factors, *Tbx21* or *Stat4*, or Th2 transcription factors, *Gata3* or *Stat6* in the brain (Fig. 4a, b). However, stroke resulted in an elevation of the Th17/γδ T cell transcription factor, *Rorc*, and this effect was mitigated by 1,25-VitD_3_ treatment (Fig. 4a). Moreover, we noted an increase in the T regulatory cell transcription factor, *Foxp3*, after stroke, and this was augmented in 1,25-VitD_3_-supplemented animals (Fig. 4b). In the spleen, 1,25-VitD_3_ had no effect on expression of *Tbx21*, *Stat4*, *Gata3*, *Stat6* or *Rorc* (Fig. 4c, d). However, *Foxp3* expression was slightly higher after stroke in 1,25-VitD_3_-treated mice than in sham mice or in those treated with vehicle (Fig. 4d).

### Effect of Vitamin D_3_ Supplementation on Expression of Pro-inflammatory Mediators in the Brain Following Stroke

**Fig. 5:**
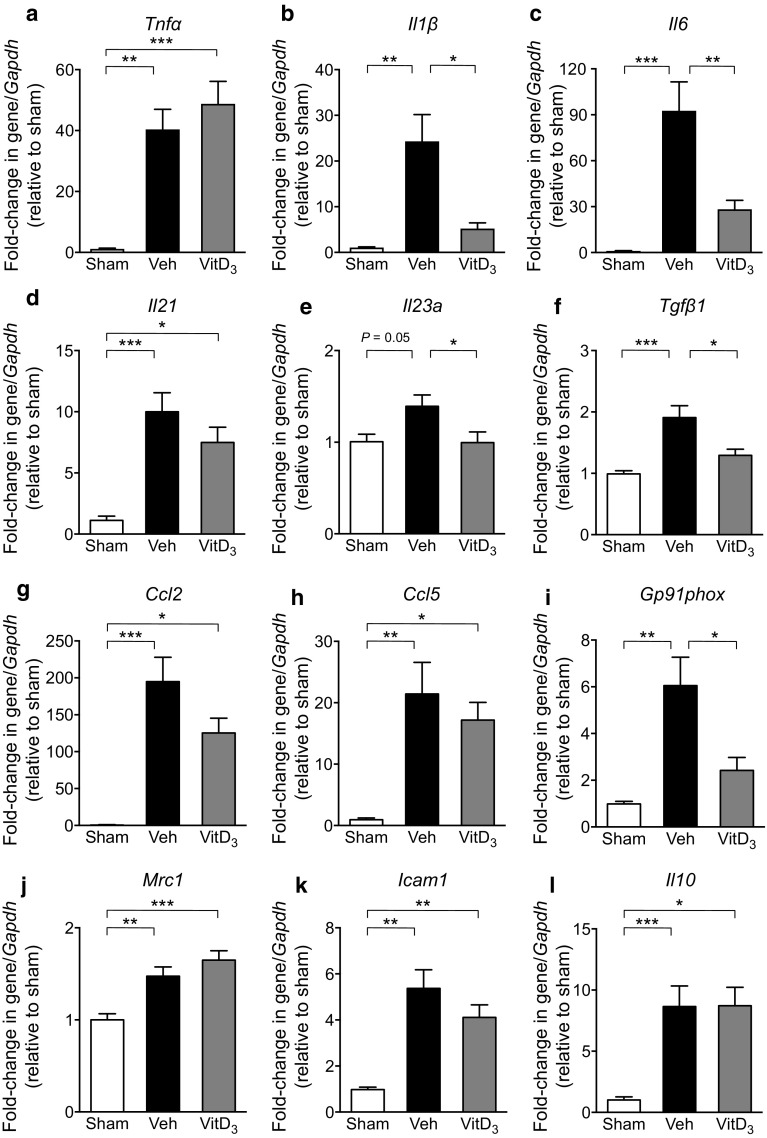
1,25-Dihydroxyvitamin D_3_ (VitD_3_) supplementation alters mRNA expression of inflammatory mediators in the brain following stroke. mRNA expression of **a** *Tnfα*, **b** *Il1β*, **c** *Il6*, **d** *Il21*, **e** *Il23a*, **f** *Tgfβ1*, **g** *Ccl2*, **h** *Ccl5*, **i** *Gp91phox*, **j** *Mrc1*, **k** *Icam1* and **l** *Il10* within the brains of sham, vehicle (Veh)- and VitD_3_-treated mice at 24 h post-surgery. Sham: *n* = 6–7 per group, vehicle: *n* = 12 per group and VitD_3_: *n* = 9–11 per group. *P < 0.05, **P < 0.01, ***P < 0.001, one-way ANOVA followed by Tukey’s multiple comparisons test. Data are presented as mean ± SEM

As vitamin D_3_ has immunomodulatory actions, we also examined mRNA expression of various inflammatory mediators known to be involved in ischemic brain injury. Indeed, 1,25-VitD_3_-treated animals had lower expression of *Ilβ*, *Il6*, *Il23a*, *Tgfβ1* and *Gp91phox* (NOX-2) than vehicle-treated animals (Fig. 5b, c, e, f, i). However, there was no effect of 1,25-VitD_3_ on *Tnfα*, *Il21*, *Ccl2*, *Ccl5*, *Mrc1*, *Icam1* or *Il10* (Fig. 5a, d, g, h, j, k, l).

### Effect of Vitamin D_3_ Supplementation on Numbers of Infiltrating Leukocytes and M2-Polarized Macrophages/Microglia in the Brain Following Stroke

**Fig. 6:**
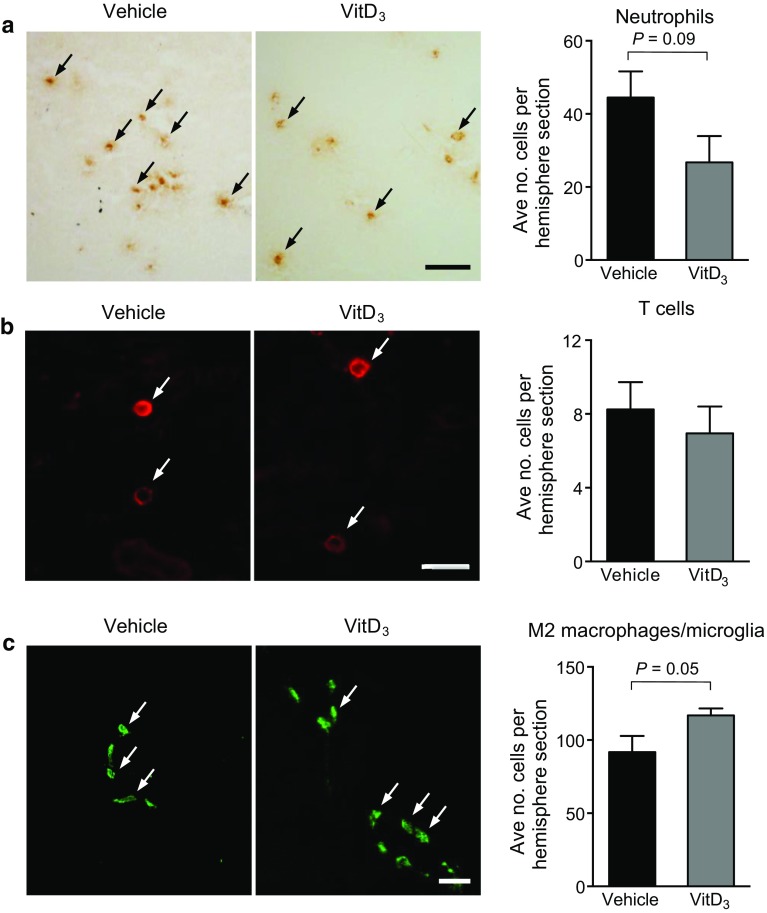
Quantification of leukocytes in the brain post-stroke. Immunohistochemistry was used to determine the numbers of **a** myeloperoxidase (MPO)^+^ cells, **b** CD3^+^ cells and **c** CD206^+^ cells per right (ischemic) hemisphere in vehicle- and 1,25-dihydroxyvitamin D_3_ (VitD_3_)-treated animals at 24-h post-stroke. Vehicle: *n* = 9–11 per group and VitD_3_: *n* = 9–10 per group. Student’s unpaired *t* test. Data are presented as mean ± SEM. Arrows on representative images indicate examples of positive cells, and scale bars represent either 20 μm (**b**) or 50 μm (**a**, **c**)

We tested for any effect of vitamin D on migration of immune cells toward the site of injury, by quantifying leukocyte infiltration into the ischemic hemisphere using immunohistochemistry. We noted a tendency for 1,25-VitD_3_-treated animals to have fewer MPO^+^ neutrophils in the brain at 24 h post-stroke, whereas there was no effect on CD3^+^ T cells (Fig. 6a, b). Additionally, there was a trend for greater numbers of “M2” polarized microglia/macrophages (defined as CD206^+^) after stroke in 1,25-VitD_3_-treated animals (Fig. 6c).

## Discussion

Inflammation is a major contributor to secondary brain injury after ischemic stroke and thus represents a potential target for therapy (Anrather and Iadecola 2016). Beyond its well-characterized role in calcium metabolism, vitamin D has potent immunomodulatory properties and can alter the immune response to injury in various disease settings (Nashold et al. 2013; Takeda et al. 2010; Martorell et al. 2016; Schedel et al. 2016). If vitamin D was found to exert such effects in post-stroke brain injury, it could represent a novel direction for acute therapy. Indeed, here we report data supporting this concept. This neuroprotective effect appears to occur in association with reduced expression of pro-inflammatory mediators in the brain. Moreover, our data suggest that 1,25-VitD_3_ supplementation alters the phenotype of T cells and increases numbers of M2 macrophages/microglia in the ischemic brain, both of which may contribute to the neuroprotection by 1,25-VitD_3_ treatment.

Previous studies have demonstrated that the VDR and the vitamin D regulatory enzymes, 1-α-hydroxylase and 24-hydroxylase, to be expressed in non-classical tissues such as the brain and activated immune cells, suggesting that vitamin D may exert paracrine functions (Penna et al. 2007; Overbergh et al. 2000; Provvedini et al. 1983; Eyles et al. 2005). Additionally, studies have documented that the expression of the VDR and these enzymes can be altered during inflammation and disease (Luo et al. 2013; Yao et al. 2015; von Essen et al. 2010; Yang et al. 2011; Liu et al. 2006; Spanier et al. 2012). In the current study, we examined expression of the VDR (*Vdr*), 1-α-hydroxylase (*Cyp27b*) and 24-hydroxylase (*Cyp24a*), in both the brain and spleen at 24 h after stroke or sham surgery. We found that expression of the VDR was elevated in both organs after stroke. Interestingly, we observed that expression of the vitamin D-activating enzyme, 1-α-hydroxylase, was reduced in the brain after stroke, while expression of the vitamin D inactivating enzyme, 24-hydroxylase, was increased. However, in the spleen, we observed that expression of 1-α-hydroxylase and 24-hydroxylase was unchanged and undetected, respectively. These findings may imply that local levels of the active form of endogenous vitamin D may be reduced in the brain after stroke, raising the possibility that supplementation with exogenous 1,25-VitD_3_ may be of benefit.

Indeed, we found that 1,25-VitD_3_-supplemented animals developed a smaller infarct volume than vehicle-treated controls by 24 h. However, at this time point, there were no apparent differences in functional outcome. While 24 h is a relatively early time point for examining outcomes after stroke, we know from our previous work that infarct size is fully developed within 24 h in this model of stroke (Evans et al. 2018). Therefore, in seeking to test whether vitamin D might exert a neuroprotective effect to limit infarct development potentially by inhibiting inflammation, we chose to examine outcomes at 24 h. However, we do acknowledge the importance of evaluating the effect of 1,25-VitD_3_ at later time points after stroke, particularly on functional recovery. Our findings are analogous to those reported by two previous studies using rat models of stroke, whereby 1,25-VitD_3_ pre-treatment reduced infarct volume (Fu et al. 2013; Oermann et al. 2004). However, neither of these studies examined functional outcome. Moreover, the precise mechanisms by which 1,25-VitD_3_ reduced brain injury in those studies were unclear.

To this end, we tested for evidence that 1,25-VitD_3_ may modulate the immune response to ischemic stroke. Vitamin D can modulate the phenotype of T cells (Hart et al. 2011; Cantorna et al. 1996). For instance, in mouse models of multiple sclerosis vitamin D can down-regulate signaling pathways essential for development of Th1 and Th17 cells (Zeitelhofer et al. 2017; Mattner et al. 2000; Muthian et al. 2006; Chang et al. 2010b; Joshi et al. 2011). Moreover, vitamin D can promote the formation of Th2 and T regulatory cells (Hart et al. 2011) and limit the development of γδ T cells (Chen et al. 2005). Several studies have revealed that Th1 and γδ T cells can aggravate brain injury after stroke, and that blocking their invasion may be neuroprotective (Gu et al. 2012; Yilmaz et al. 2006; Gelderblom et al. 2012; Shichita et al. 2009). Th2 and T regulatory cells are thought to be injury-limiting in the setting of stroke (Gu et al. 2012; Liesz et al. 2009). We thus examined whether the neuroprotection by 1,25-VitD_3_ may be associated with modulation of T cell phenotypes. In the brain, we found that neither stroke nor 1,25-VitD_3_ had any effect on expression of Th1 or Th2 transcription factors. However, 1,25-VitD_3_ blunted expression of the Th17/γδ T cell transcription factor, *Rorc* (ROR-γt), and enhanced expression of the T regulatory cell transcription factor, *Foxp3*. In the spleen 1,25-VitD_3_ increased expression of *Foxp3*, but had no effect on Th1, Th2 or Th17/γδ transcription factors. Collectively, these data may indicate that 1,25-VitD_3_ promotes the formation of T regulatory cells while inhibiting development of Th17/γδ T cells, consistent with a neuroprotective profile.

1,25-VitD_3_ supplementation reduced mRNA expression of pro-inflammatory cytokines, *Il1β* (IL-1β), *Il6* (IL-6), *Tgfβ1* (TGF-β) and *Il23a* (IL-23a). Interestingly, these cytokines are thought to play key roles in the function of both Th17 and γδ T cells (Vantourout and Hayday 2013). Treatment with 1,25-VitD_3_ had no effect on expression of *Il10* (IL-10), an immunosuppressive cytokine often involved in T regulatory cell function (Taylor et al. 2006); however, T regulatory cells may limit injury and excessive inflammation via other mechanisms (Sakaguchi et al. 2009). We also observed a reduction in *gp91phox* (NOX2) expression, a key producer of superoxide and mediator cellular damage following ischemic stroke (De Silva et al. 2011).

As 1,25-VitD_3_ can reduce leukocyte recruitment to injured tissues (Pedersen et al. 2007; Korf et al. 2012; Grishkan et al. 2013), we examined its effect on leukocyte infiltration into the brain following stroke. We observed a trend for 1,25-VitD_3_-treated animals to have fewer infiltrating neutrophils but no apparent effect on T cells. It is also possible that 1,25-VitD_3_ reduces recruitment of other types of immune cell subsets or that it modulates their functional phenotype rather than migration to the site of post-stroke injury. It is important to note that 24 h represents a relatively early pathological time point after stroke with significant immune cell infiltration continuing after this time point (Gelderblom et al. 2009; Benakis et al. 2016). We also examined the effect of 1,25-VitD_3_ on the numbers of M2-polarized macrophages/microglia in the brain after stroke. Studies have reported that M2 macrophages/microglia are likely to be protective in the setting of stroke by reducing inflammation and coordinating repair processes (Benakis et al. 2014; Chu et al. 2015; Hu et al. 2012). We observed a strong trend for 1,25-VitD_3_ to augment numbers of CD206^+^ M2 macrophages/microglia in the brain after stroke.

As mentioned above, there is a strong rationale to gain a deeper understanding of how altered levels of vitamin D—prior and/or subsequent to stroke—might impact on the degree of ensuing brain injury. Following on from our previous finding that low baseline vitamin D levels did not impact on outcome measures at 24 h (Evans et al. 2017), here we have instead assessed the effect of increasing baseline levels achieved by five daily supraphysiological doses of vitamin D prior to stroke. Indeed, the present data suggest that supplementing mice with the active form of vitamin D prior to stroke can reduce the extent of brain injury. With regard to therapeutic relevance for acute clinical stroke, our data are important in terms of proof-of-concept. However, a limitation is that 1,25-VitD_3_ was administered only prior to stroke induction, and clearly, studies are now required in which post-stroke treatment of 1,25-VitD_3_ is evaluated. While our data suggest that 1,25-VitD_3_ can also modulate the immune response to brain injury following stroke, at least part of this protection may occur via non-immune mechanisms, such as inhibiting excitotoxicity (Taniura et al. 2006; Brewer et al. 2001), stimulating production of neurotrophic factors (Neveu et al. 1994; Naveilhan et al. 1996; Landel et al. 2016) or improving blood brain barrier integrity (Won et al. 2015). It is also noteworthy that we administered 1,25-VitD_3_ to mice that were vitamin D replete. Whether a similar or greater level of neuroprotection might be achieved in vitamin D-deficient animals by 1,25-VitD_3_ therapy will be important to clarify.

In conclusion, these findings indicate that administration of vitamin D can attenuate infarct development following stroke possibly by modulating the inflammatory response to cerebral ischemia. Therefore, vitamin D supplementation may represent a novel direction for limiting the impact of acute stroke.

## Electronic Supplementary Material

Below is the link to the electronic supplementary material.

Supplementary material 1 Cerebral blood flow profile. Regional cerebral blood flow was recorded during and after 1 h of middle cerebral artery occlusion in mice, treated either with vehicle (Veh) or 1,25-dihydroxyvitamin D_3_ (VitD_3_). Vehicle: *n* = 20 per group, VitD_3_: *n* = 20 per group. Data are presented as mean ± SEM
