# Supplementary figures and images for: Vitamin D3 Supplementation Reduces Subsequent Brain Injury and Inflammation Associated with Ischemic Stroke
Source: Neuromolecular Med. 2018 Feb 23;20(1):147–59. doi: 10.1007/s12017-018-8484-z (PMC5834596; doi:10.1007/s12017-018-8484-z)

## Slide 1
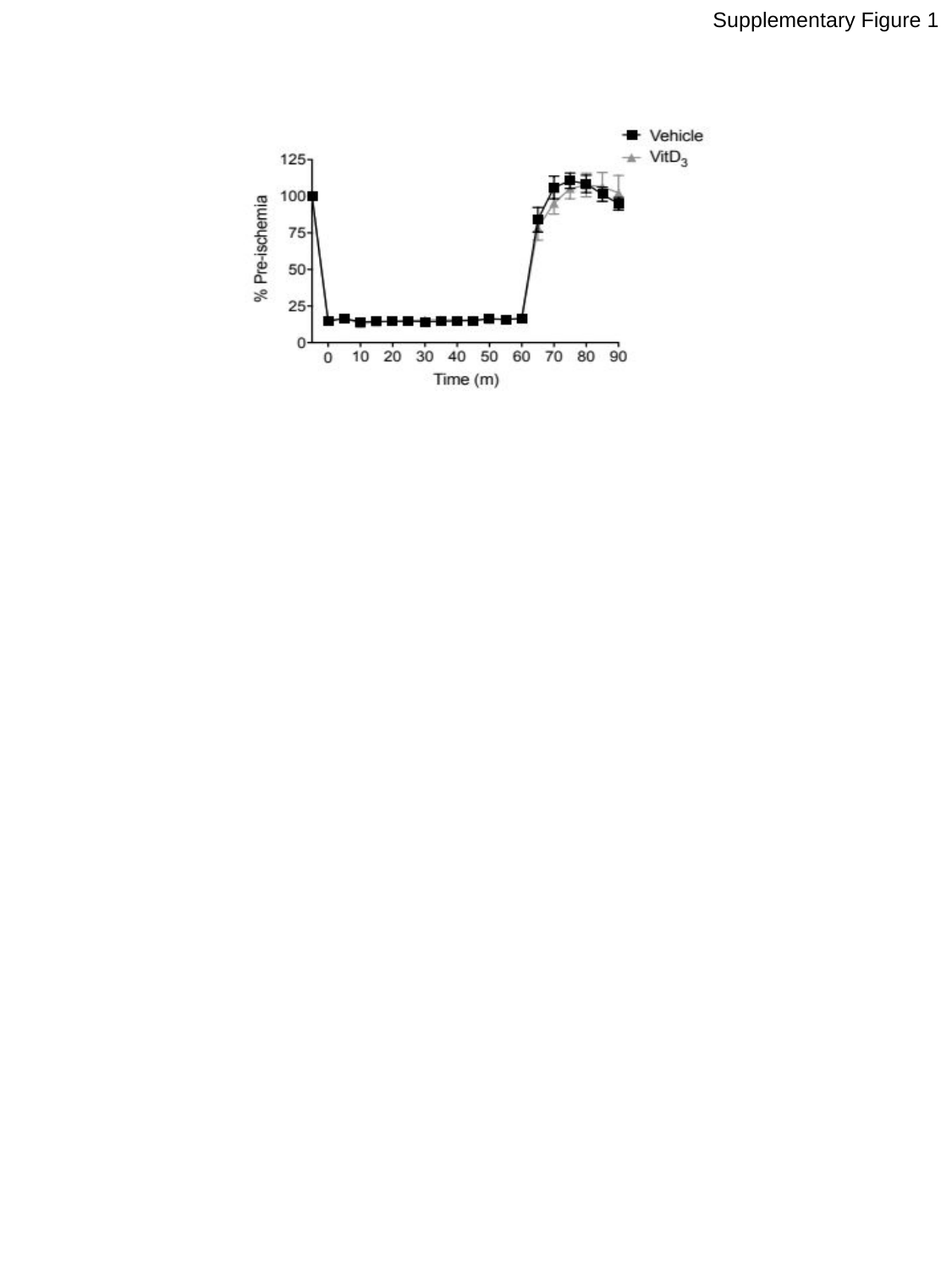

Supplementary Figure 1

Supplement: Supplementary file 1 — Supplementary material 1 Cerebral blood flow profile. Regional cerebral blood flow was recorded during and after 1 h of middle cerebral artery occlusion in mice, treated either with vehicle (Veh) or 1,25-dihydroxyvitamin D3 (VitD3). Vehicle: n = 20 per group, VitD3: n = 20 per group. Data are presented as mean ± SEM [file 12017_2018_8484_MOESM1_ESM.pptx]
